# The correlation between sarcopenia and osteoporosis in the elderly: a systematic review and meta-analysis

**DOI:** 10.3389/fmed.2025.1603879

**Published:** 2025-10-01

**Authors:** Sha Jin, Fengming Zheng, Huaili Liu, Luping Liu, Jie Yu

**Affiliations:** Department of Geriatrics, Hangzhou Third People’s Hospital, Hangzhou, China

**Keywords:** sarcopenia, osteoporosis, correlation, systematic review, meta-analysis

## Abstract

**Background:**

Sarcopenia and osteoporosis, as two prevalent geriatric syndromes, synergistically elevate risks of falls, fractures, and disability in older adults. Despite shared pathophysiological mechanisms–including hormonal dysregulation, chronic inflammation, and attenuated mechanical loading. Existing studies have yet to establish consensus regarding the epidemiological association strength and interaction dynamics between sarcopenia and osteoporosis, particularly as heterogeneous characteristics–including sex, geographic region, and population subgroups–remain insufficiently characterized. This study aimed to quantitatively evaluate the sarcopenia-osteoporosis association in older adults through systematic review and meta-analysis of global observational studies, while analyzing the moderating effects of geographic location, sex, population characteristics, and diagnostic criteria on outcomes.

**Methods:**

We searched PubMed, Embase, Cochrane Library, and China National Knowledge Infrastructure (CNKI) databases until September 2024. Fourteen observational studies quantifying muscle mass/function and bone mineral density were included. Two investigators independently performed literature screening and data extraction. Study quality was assessed using the Newcastle-Ottawa Scale (NOS). Studies were meta-analyzed by Review Manager 5.4 and Stata 17.0.

**Results:**

A total of 14 studies (*n* = 182307) were included, and the meta-analysis showed that patients with sarcopenia had a significantly higher risk of osteoporosis (OR = 3.16, 95% CI: 2.47 to 4.02, *p* < 0.001). Subgroup analyses demonstrated an OR of 4.74 [3.19, 7.06] for osteoporosis in the male sarcopenia group compared to females (OR = 3.46; 95% CI, 2.50–4.78). Geographically, European populations exhibited the highest risk (OR = 4.37; 95% CI, 3.72–5.13), surpassing Asian (OR = 2.66; 95% CI, 1.74–4.07) and American cohorts (OR = 2.32; 95% CI, 1.54–3.49). Community-dwelling individuals showed greater susceptibility (OR = 3.70; 95% CI, 3.24–4.23) compared to inpatient and outpatient populations.

**Conclusion:**

Our meta-analysis demonstrates that sarcopenia significantly elevates osteoporosis susceptibility, with heterogeneous risk profiles across geographic regions and population subgroups. However, limitations inherent to the methodological quality and sample size of included studies necessitate validation through large-scale prospective cohort investigations.

## 1 Introduction

The global demographic shift toward aging populations has brought increasing attention to geriatric health challenges. Sarcopenia and osteoporosis, two prevalent geriatric syndromes, manifest as progressive declines in muscle mass and bone mineral density respectively. Epidemiological data indicate a sarcopenia prevalence of 10%–27% among individuals aged ≥60 years (1), while osteoporosis affects 30%–50% of this population (2). Emerging evidence reveals significant pathophysiological intersections and clinical synergies between these conditions, warranting systematic investigation.

From an etiological perspective, shared risk mediators include senescence-associated biological processes, hormonal alterations (particularly in estrogen, androgen, and growth hormone levels), nutritional insufficiencies (notably protein, vitamin D, and calcium deficiencies), chronic low-grade inflammation, and reduced physical activity (3). Clinically, these conditions exhibit bidirectional progression: sarcopenia-induced muscle weakness impairs osteogenic mechanical loading, accelerating bone density loss, while osteoporosis-related pain and fracture risk exacerbate mobility limitations, thereby perpetuating musculoskeletal deterioration.

Despite growing research interest, current understanding remains constrained by methodological heterogeneity across studies. Discrepancies in diagnostic criteria, population characteristics, and assessment protocols have yielded inconsistent findings regarding the magnitude and mechanisms of sarcopenia-osteoporosis associations. This knowledge gap underscores the imperative for rigorous evidence synthesis through systematic review and meta-analysis. Such methodology enables quantification of correlation strength, identification of heterogeneity sources, and elucidation of potential pathophysiological convergences.

This study intends to comprehensively collect studies on the correlation between sarcopenia and osteoporosis in older adults through systematic evaluation and meta-analysis. Literature was strictly screened, data were extracted and quality evaluated from studies that met the inclusion criteria, and meta-analysis was performed using appropriate statistical models, aiming at clarifying the degree of correlation between the two in the elderly population, further exploring their potential common pathogenic mechanisms and influencing factors, and providing a more reliable basis for early clinical diagnosis, prevention and comprehensive treatment, so as to improve the health status and quality of life of the elderly.

## 2 Data and methods

### 2.1 Literature search strategy

Two researchers independently searched PubMed, Embase, Cochrane Library, and China National Knowledge Infrastructure (CNKI), spanning from their respective inceptions to September 1, 2024. The search terms were “sarcopenia,” “osteoporosis,” and “elderly.”

### 2.2 Inclusion and exclusion criteria

Studies were included if they fulfilled the following conditions: ① the type of study was observational design (covering cross-sectional, case-control or cohort studies); ② Sarcopenia was defined as the primary exposure variable, and the control group consisted of individuals with normal skeletal muscle function. ③ Osteoporosis was clearly defined as the primary outcome measure. ④ Extractable effect size data (e.g., OR value and 95% CI) or dichotomous outcome indicators were provided. The definition of sarcopenia should be based on established criteria, such as those proposed by the Asian Working Group for Sarcopenia (AWGS), the European Working Group on Sarcopenia in Older People (EWGSOP), or the skeletal muscle mass index (SMI). Osteoporosis diagnosis should be based on well - recognized methods, preferably dual - energy X - ray absorptiometry (DXA) according to the World Health Organization (WHO) criteria.

Exclusion criteria comprised: ① repetitive literature; ② non-clinical studies (e.g., animal experiments, reviews, case reports and Meta-analysis); ③ disease-specific subgroup studies (e.g., patients with renal failure); ④ literature with missing data or unable to obtain complete information.

### 2.3 Literature extraction and quality evaluation

Two researchers executed the search process separately, independently screened the literature, extracted data, and cross-validated the data based on the pre-set inclusion and exclusion criteria. In case of disagreement, a group discussion or a third-party expert (Liu Luping) was asked to intervene for a decision. The extracted information covered the following key parameters: first author, year of publication, sample size, gender distribution, geographical area of study, population type (e.g., community-dwelling, inpatients, outpatients), diagnostic criteria for sarcopenia, odds ratios (ORs) for osteoporosis.

We assessed the methodological quality using the Newcastle-Ottawa Scale (NOS) (4), which scores three dimensions (total score of 9): study population selection, between-group comparability, and assessment of outcomes. The assessment was done independently by two researchers, Jin Sha and Zheng Fengming, and reviewed by a third person (Liu Luping). The risk of bias was categorized into three levels based on the total score: high risk (<5 points), medium risk (6–7 points), and low risk (8–9 points).

### 2.4 Statistical analysis

All statistical analyses were done by Review manager 5.4 and Stata 17.0 software. Inter-study heterogeneity was assessed by the Q-test I^2^ statistic. If I^2^ was ≥50% or *P* < 0.05, a random-effects model was selected; conversely, a fixed-effects model was used. Subgroup analyses were performed to explore the causes of heterogeneity. In addition, publication bias was assessed using funnel plots. If asymmetry was found, Egger’s test was performed (significance level set at *p* less than 0.05).

## 3 Results

### 3.1 Literature search process and results

**FIGURE 1:**
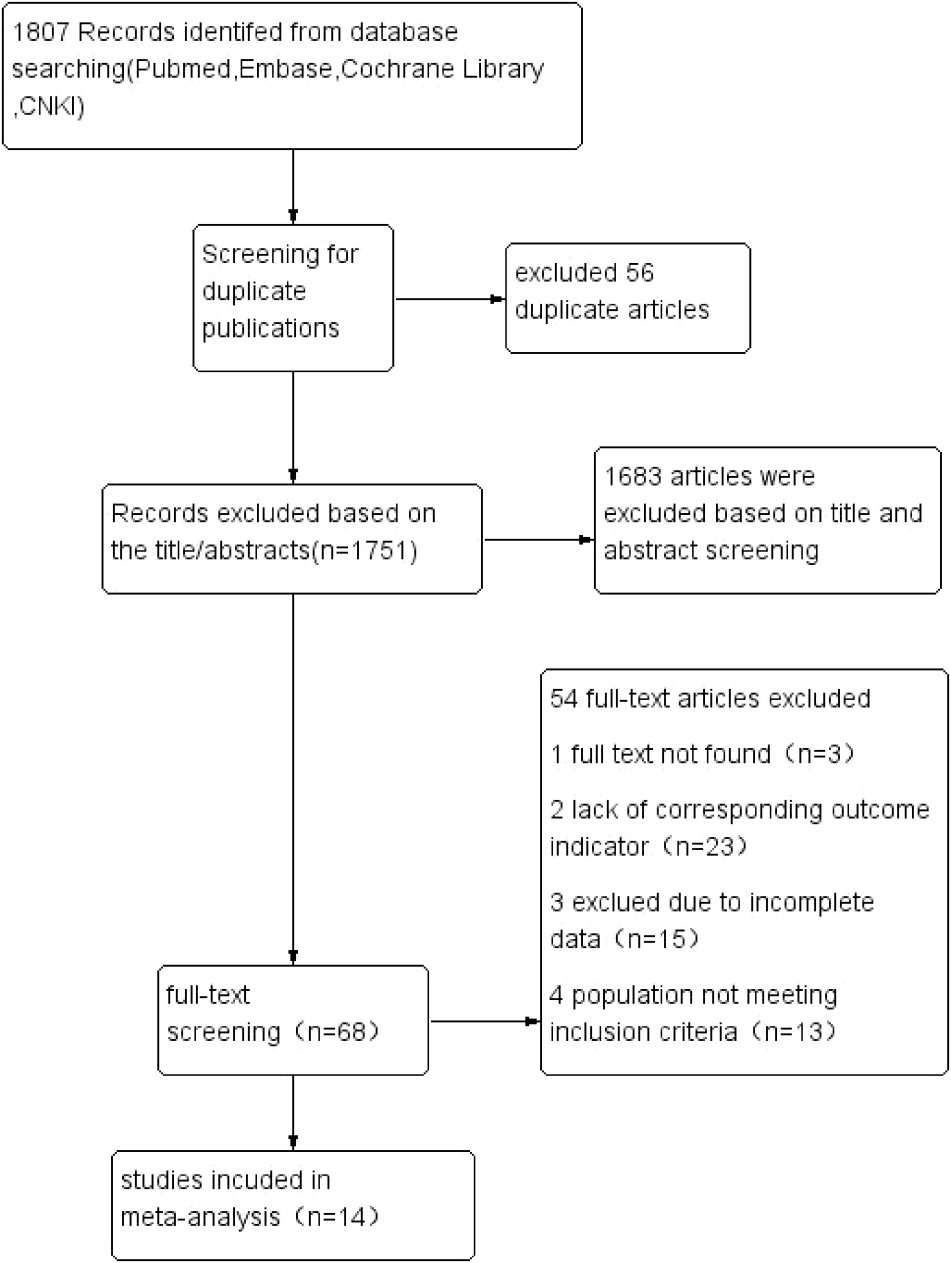
PRISMA flowchart of literature screening.

The systematic search identified 1,807 articles across PubMed, Embase, Cochrane Library, and China National Knowledge Infrastructure (CNKI). After removal of 56 duplicates, 1,751 records underwent title/abstract screening, yielding 68 potentially eligible studies. Following full-text review and application of inclusion criteria, 14 observational studies (5–18) were ultimately included for meta-analysis. The final cohort comprised 10 cross-sectional studies and 4 prospective cohort studies, encompassing 182,307 individuals with sarcopenia. Figure 1 illustrates the PRISMA-compliant selection flowchart.

### 3.2 Basic characteristics of the included literature

The baseline characteristics of the included studies are summarized in Table 1. This meta-analysis pooled data from 182,307 participants (92,073 males; 90,234 females), comprising 2,641 sarcopenic cases and 179,666 non-sarcopenic controls. The studies were conducted across diverse geographical regions spanning Asia, Europe, and the Americas. With respect to study populations, 9 investigations focused on community-dwelling individuals (5–7, 10, 11, 13, 14, 17, 18), 3 studies involved hospitalized patients (9, 12, 15), and 2 reports examined outpatient cohorts (8, 16).

**TABLE 1:** Characteristics of included studies.

Study	Region	Study design	Population type	Mean age (years)	Sample size	Sex (m/f)	Sarcopenia diagnostic criteria	Sarcopenia (yes/no)	Osteoporosis (yes/no)
**Main characteristics of studies included in the meta-analysis**									
Verschueren et al. (5)	Europe (UK, Belgium)	Cross-sectional	Community-dwelling	59.6	679	679/0	SMI	80/599	60/619
Locquet et al. (6)	Europe (Belgium)	Cross-sectional	Community-dwelling	74.7	288	118/170	EWGSOP	43/245	36/252
Trajanoska et al. (7)	Europe (Netherlands, Belgium)	Prospective cohort	Community-dwelling	69.2	5,911	2,613/3,298	EWGSOP	47/5864	278/5586
Ontan et al. (8)	Europe (Turkey)	Prospective cohort	Outpatients	75.88	444	85/359	EWGSOP-2	133/311	144/300
Pourhassan et al. (9)	Europe (Germany)	Prospective cohort	Inpatients	75.1	572	126/445	EWGSOP-2	52/520	190/382
Yoshimura et al. (10)	Asia (Japan)	Prospective cohort	Community-dwelling	72.1	1,099	377/722	AWGS	90/1009	273/826
Lee and Shin (11)	Asia (South Korea)	Cross-sectional	Community-dwelling	71.82	3,077	1,376/1,701	AWGS	1230/1847	1193/1884
Di Monaco et al. (12)	Europe (Italy)	Cross-sectional	Inpatients	79.7	262	0/262	EWGSOP-2	147/115	189/73
Petermann-Rocha et al. (13)	Europe (UK)	Cross-sectional	Community-dwelling	56.2	168,682	86,385/82,297	EWGSOP-2	559/68123	6292/162390
Lima et al. (14)	Americas (Brazil)	Cross-sectional	Community-dwelling	68.3	234	0/234	EWGSOP	43/191	46/188
Reiss et al. (15)	Europe (Austria)	Cross-sectional	Inpatients	80.6	141	57/84	EWGSOP	39/102	42/99
Frisoli et al. (16)	Americas (Brazil)	Cross-sectional	Outpatients	78.44	332	141/191	EWGSOP	64/268	117/215
Kuriyama et al. (17)	Asia (Japan)	Cross-sectional	Community-dwelling	77.2	321	116/205	AWGS	73/248	92/229
Taniguchi et al. (18)	Asia (Japan)	Cross-sectional	Community-dwelling	75.5	265	0/265	SMI	131/134	72/193

SMI, skeletal muscle index; AWGS, Asian Working Group for Sarcopenia; EWGSOP, European Working Group on Sarcopenia in Older People.

3 studies employed the Asian Working Group for Sarcopenia (AWGS) diagnostic criteria (10, 11, 17), while 5 investigations applied the European Working Group on Sarcopenia in Older People (EWGSOP) operational definitions (6, 7, 14–16). 2 studies adopted skeletal muscle mass index (SMI) thresholds (5, 18), and 4 implemented the updated EWGSOP-2 consensus criteria (8, 9, 12, 13).

Methodological quality assessment using the Newcastle-Ottawa Scale (NOS) revealed robust study design across all included publications. Each study achieved a NOS score ≥7, indicating high methodological quality in terms of participant selection, comparability assessment, and outcome ascertainment.

### 3.3 Meta-analysis results

**FIGURE 2:**
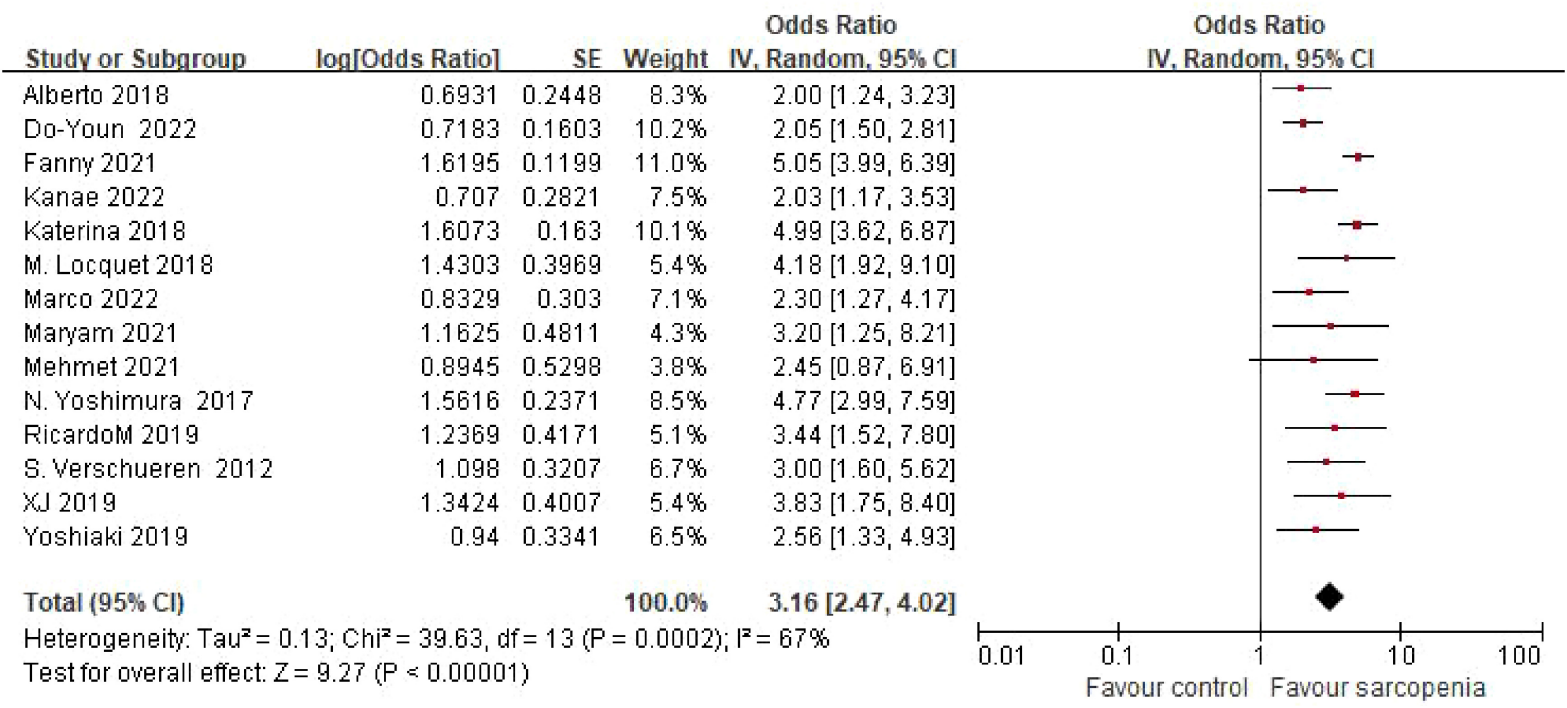
Forest plot of odds ratio (OR) for osteoporosis risk in sarcopenic vs. non - sarcopenic based on Meta – analysis.

The systematic review incorporated 14 observational studies evaluating the sarcopenia-osteoporosis association. Seven investigations (5, 6, 8, 11, 12, 14, 18) reported odds ratios (ORs) with 95% confidence intervals (CIs) for the sarcopenia-osteoporosis association, while seven studies (7, 9, 10, 13, 15–17) provided dichotomous outcome data. Given substantial heterogeneity among studies (I^2^ = 67%, *P* < 0.05), a random-effects model was employed. Pooled analysis revealed a 3.16-fold (OR = 3.16, 95% CI: 2.47–4.02; *P* < 0.001) increased osteoporosis risk in sarcopenic individuals (*n* = 2641) compared to non-sarcopenic controls (*n* = 179,666) (Figure 2). Subgroup analyses were also performed to explore sources of heterogeneity.

### 3.4 Subgroup analyses

#### 3.4.1 Sex-specific risk gradients

**FIGURE 3:**
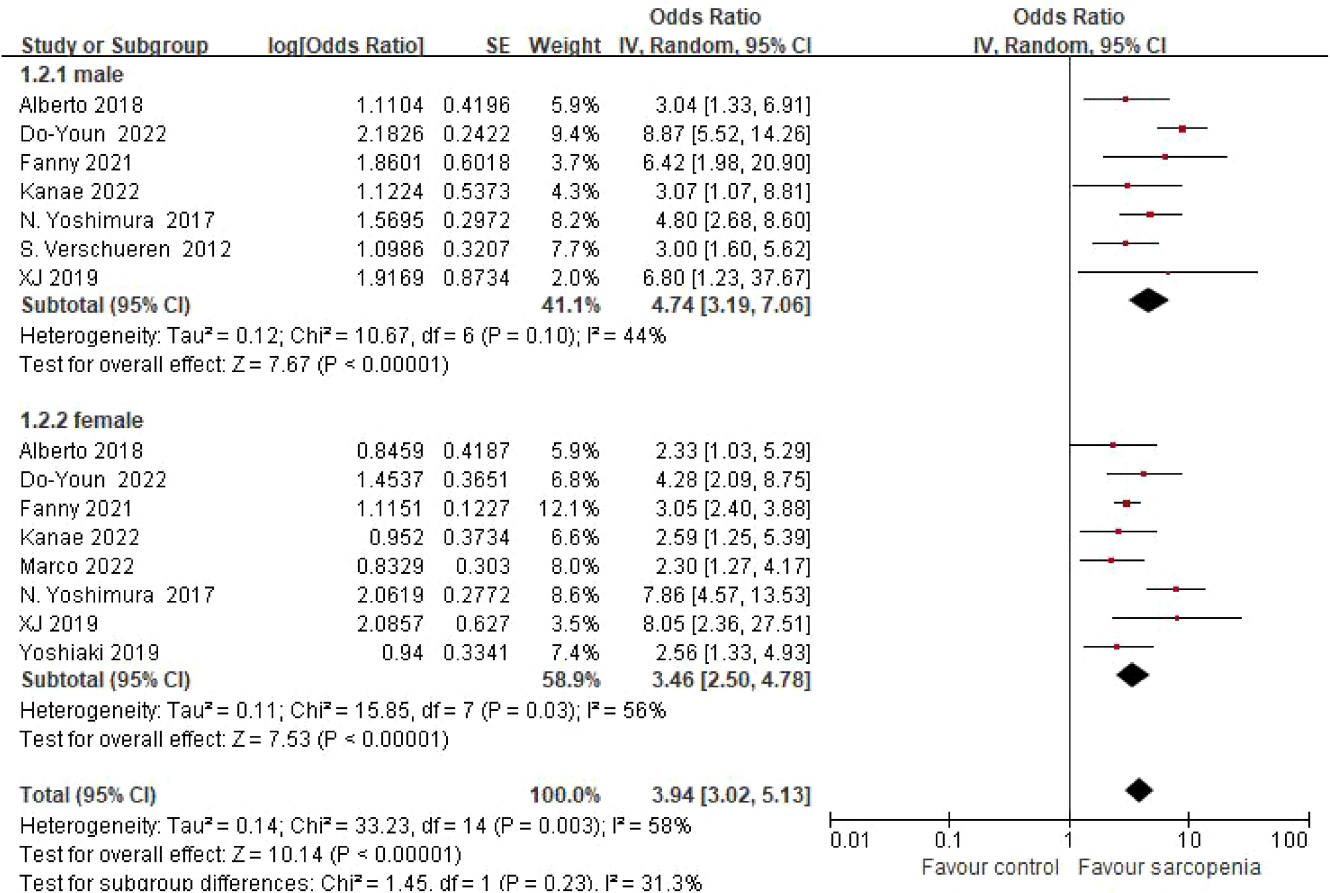
Meta-analysis forest plot of osteoporosis risk ratio (OR) in sarcopenic vs. non-sarcopenic subjects across gender subgroups.

Sex-stratified meta-analysis revealed pronounced sex-specific risk gradients (Figure 3). Pooled estimates from 7 male-only cohorts demonstrated a 4.74-fold increased osteoporosis risk in sarcopenic males (OR = 4.74, 95% CI: 3.19–7.06). Conversely, female participants (8 studies) exhibited a 3.46-fold risk elevation (OR = 3.46, 95% CI: 2.50–4.78). No statistically significant sex-based interaction was detected (*P* = 0.23), though male effect magnitudes were numerically greater.

#### 3.4.2 Geographic disparities in risk estimates

**FIGURE 4:**
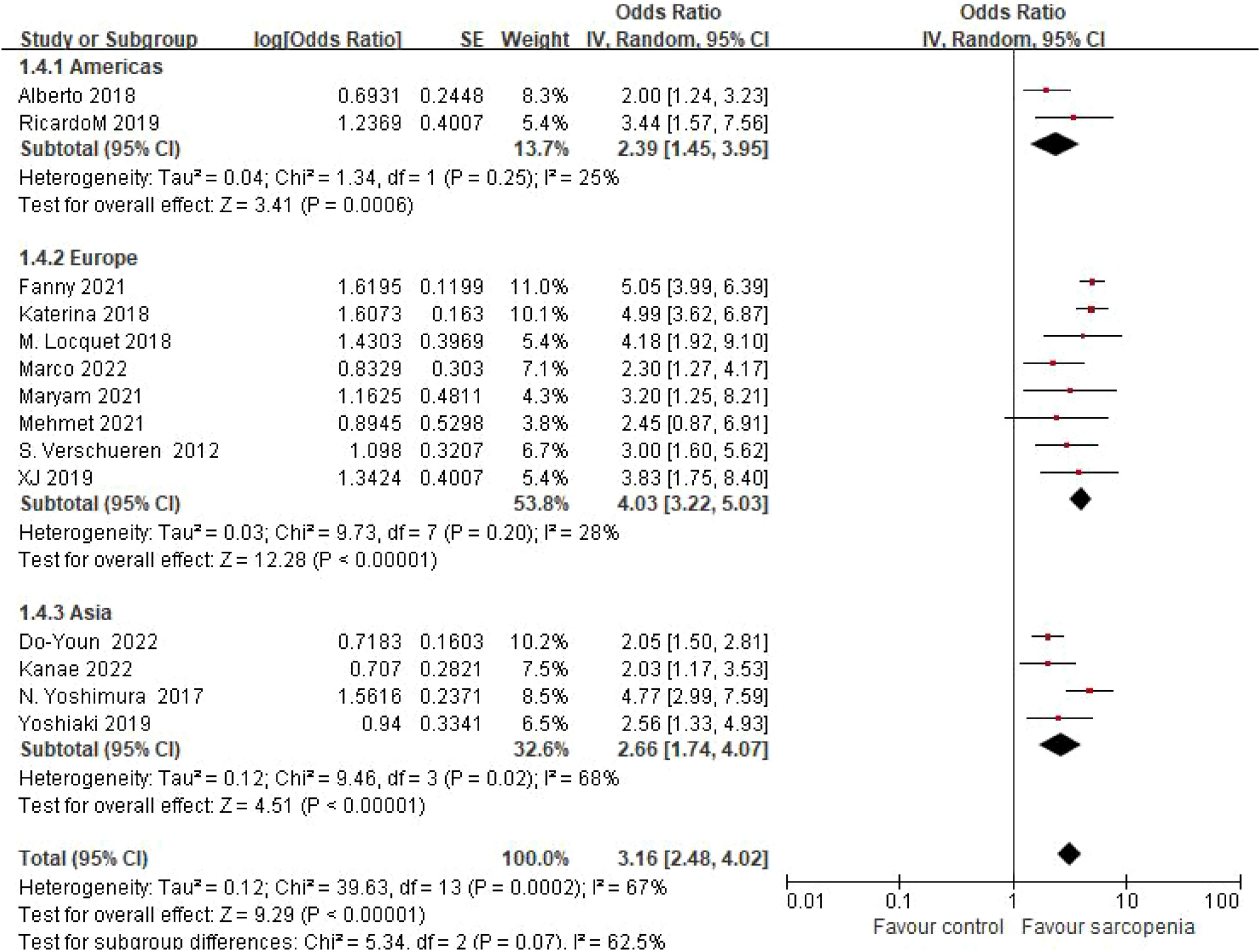
Forest plot of osteoporosis risk ratio (OR) in sarcopenia vs. non - sarcopenia patients by geographic regions.

Eight studies originated from Europe, two from North America, and four from Asia. Pooled estimates revealed odds ratios (ORs) of 4.74 (95% CI: 3.19–7.06) for European cohorts, 2.66 (95% CI: 1.74–4.07) for Asian populations, and 2.32 (95% CI: 1.54–3.49) for North American groups. No statistically significant differences were observed between geographic subgroups (*P* = 0.07) (Figure 4).

#### 3.4.3 Healthcare setting-specific associations

**FIGURE 5:**
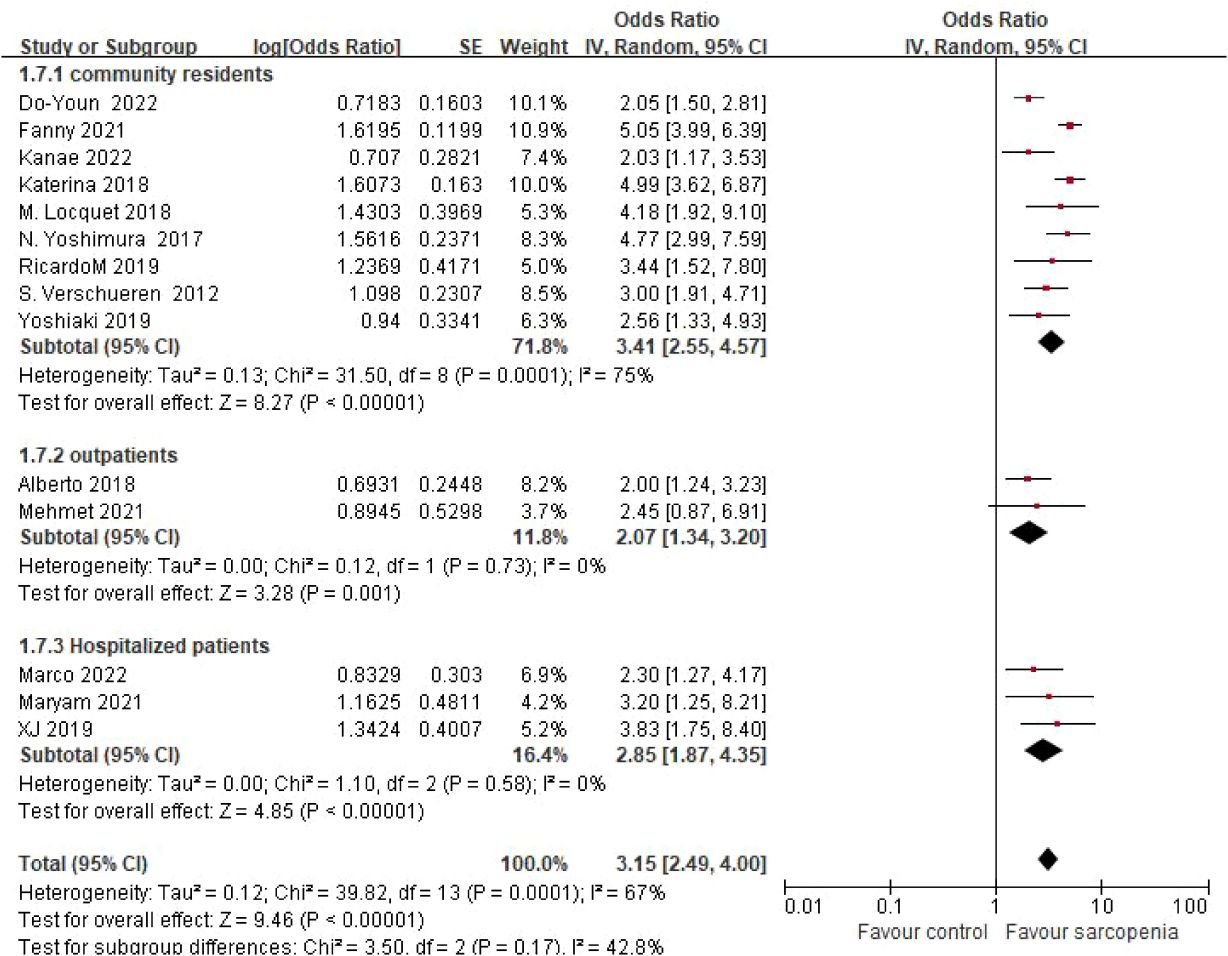
Forest plot of osteoporosis risk ratio (OR) in sarcopenia vs. non - sarcopenia patients by healthcare settings.

Subgroup analysis by participant setting showed marked differences in risk estimates. Community-dwelling populations (9 studies) had the highest osteoporosis risk (OR = 3.41, 95% CI: 2.55–4.57), followed by inpatients (2 studies, OR = 2.85, 95% CI: 1.87–4.35) and outpatients (3 studies, OR = 2.07, 95% CI: 1.34–3.20). No statistically significant differences were observed between healthcare settings (*P* = 0.17) (Figure 5).

#### 3.4.4 Grouping according to diagnostic criteria

**FIGURE 6:**
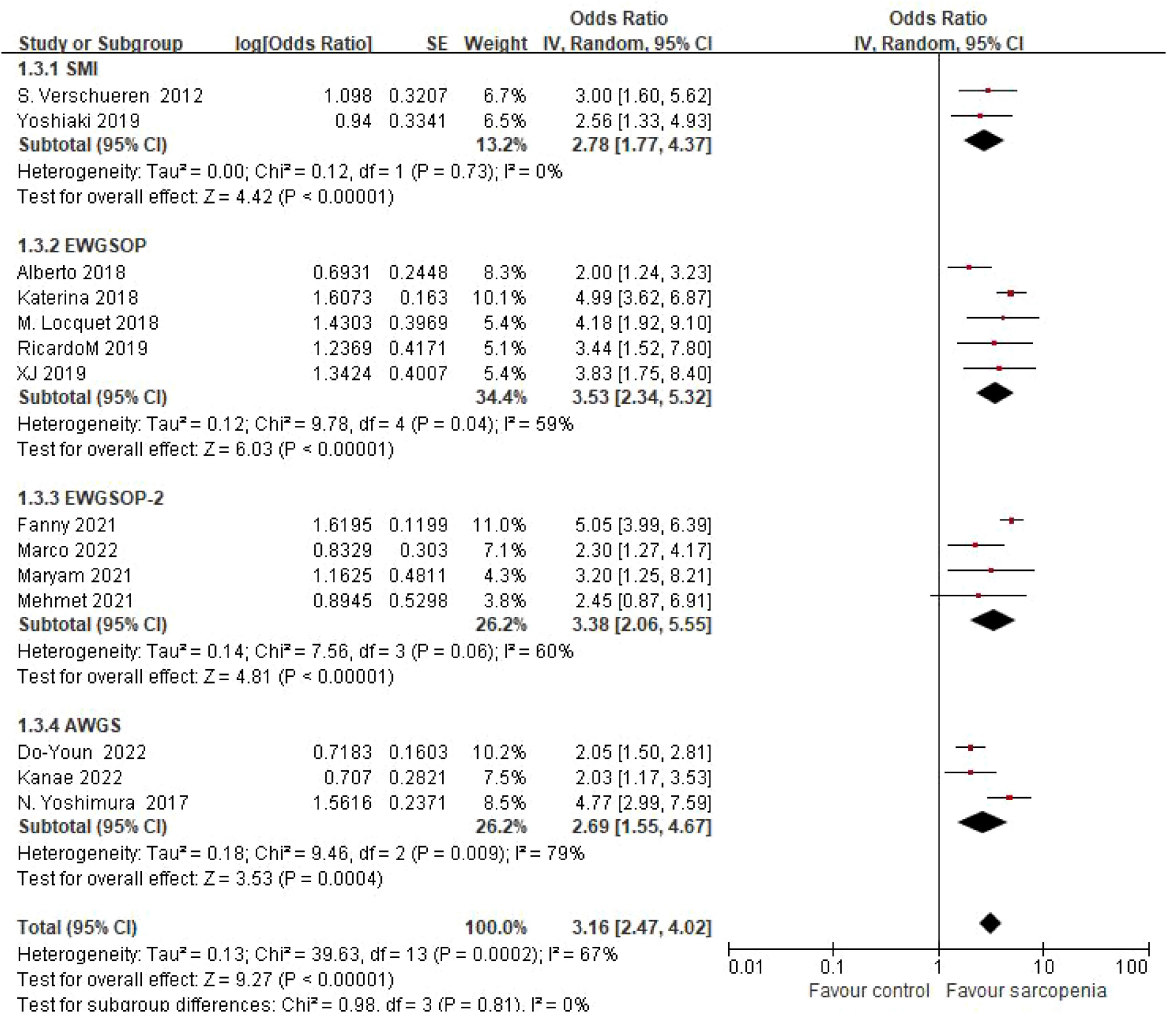
Forest plot comparing osteoporosis OR between sarcopenic and non - sarcopenic groups by diagnostic criteria.

Three studies using Asian Working Group for Sarcopenia (AWGS) criteria demonstrated an OR of 2.69 (95% CI: 1.55–4.67), while five studies applying European Working Group on Sarcopenia in Older People (EWGSOP) criteria yielded an OR of 3.53 (95% CI: 2.34–5.32). Four studies implementing updated EWGSOP-2 criteria reported an OR of 3.38 (95% CI: 2.06–5.55), and two studies using skeletal muscle mass index (SMI)-based thresholds showed an OR of 2.78 (95% CI: 1.77–4.37). Subgroup analysis by diagnostic criteria revealed consistent risk estimates across definitions. No statistically significant differences were observed between diagnostic subgroups (*P* = 0.81) (Figure 6).

#### 3.4.5 Grouping according to study design

**FIGURE 7:**
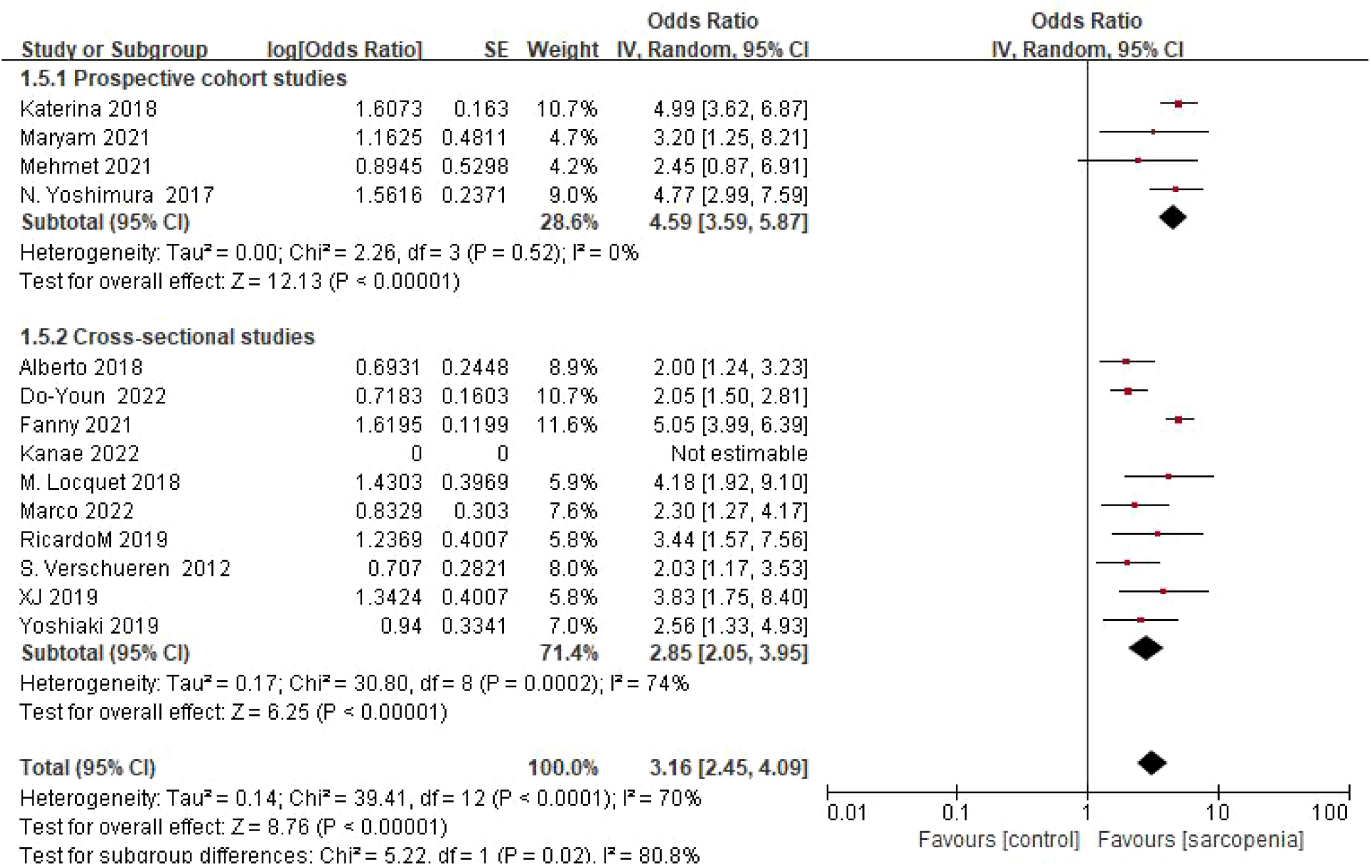
Forest plot comparing osteoporosis OR between prospective cohort studies and cross-sectional studies.

Four cohort studies showed an OR of 4.59 (95% confidence interval: 3.59–45.87), while 10 cross-sectional studies indicated an OR of 2.85 (95% confidence interval: 2.05–3.95). Subgroup analysis grouped by study design revealed consistent risk estimates across different study types. There was a statistically significant difference between subgroups (*P* = 0.02) (Figure 7).

### 3.5 Publication bias

**FIGURE 8:**
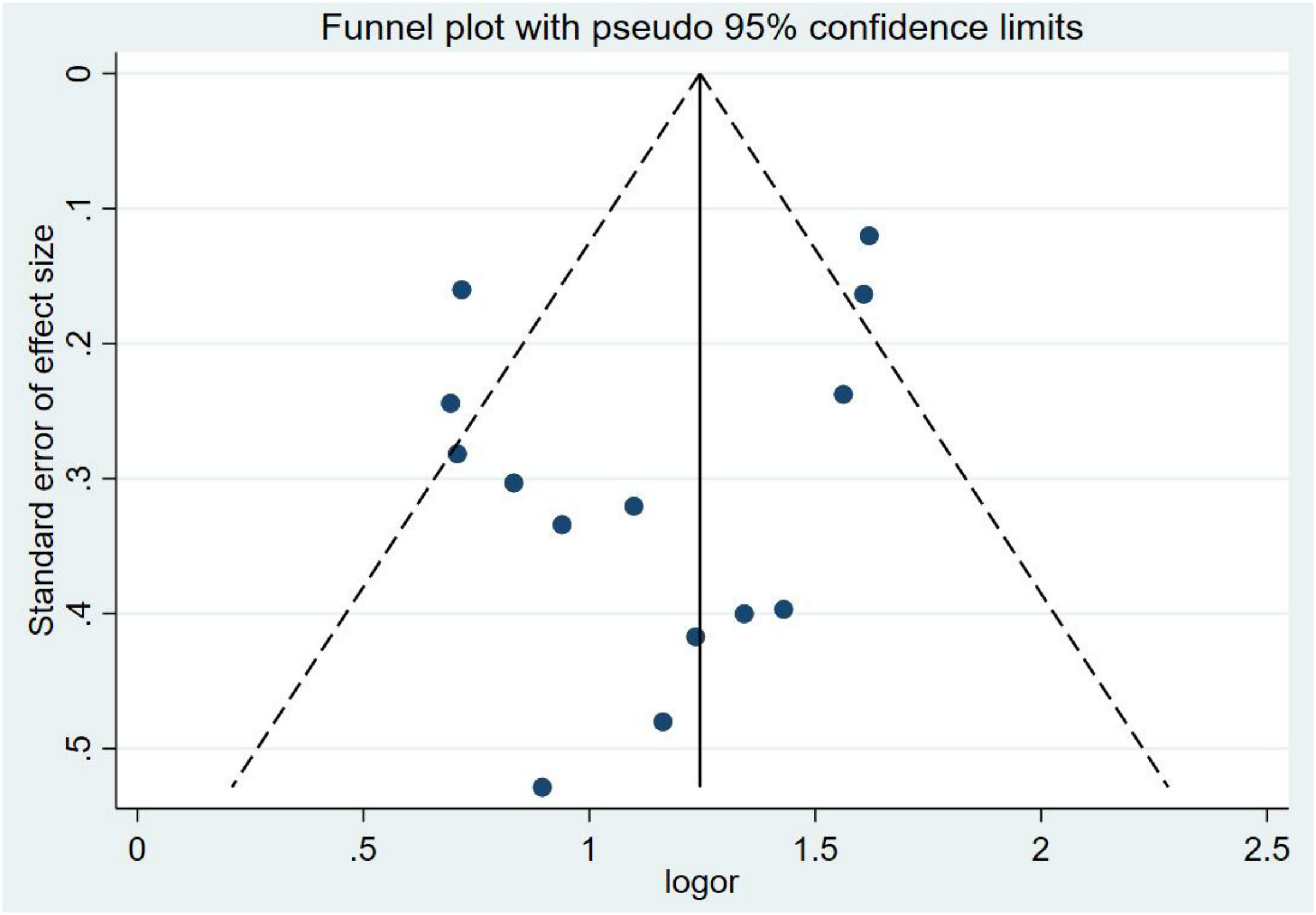
Funnel plot of publication bias.

Publication bias was evaluated using funnel plot symmetry and Egger’s regression test. Visual inspection of the funnel plot (Figure 8) revealed approximate symmetry, with no obvious asymmetry suggesting small-study effects. Egger’s regression analysis demonstrated no statistically significant publication bias (*P* = 0.20), confirming the absence of systematic bias in the included studies.

## 4 Discussion

This meta-analysis of 14 studies (*n* = 182,307) provides the first systematic quantification of the sarcopenia-osteoporosis association, demonstrating a 3.16-fold increased osteoporosis risk in sarcopenic individuals compared to controls (OR = 3.16, 95% CI: 2.47–4.02). These findings underscore the pathophysiological synergy between these geriatric syndromes, likely mediated by shared mechanisms including hormonal dysregulation (e.g., IGF-1 deficiency, vitamin D insufficiency) and chronic inflammation (19).

Notably, subgroup analyses revealed sex-specific trends: males showed numerically higher risk (OR = 4.74) compared to females (OR = 3.46), though interaction testing was non-significant (*P* = 0.23), potentially reflecting estrogen’s protective effects on female bone health. Geographic disparities emerged, with European populations demonstrating numerically higher risk (OR = 4.74) than other subgroups (*P* = 0.07), possibly due to genetic predispositions and lifestyle factors (e.g., protein intake patterns). Community-dwelling individuals also exhibited greater osteoporosis risk (OR = 3.41) compared to inpatients (OR = 2.85) and outpatients (OR = 2.07), which may reflect earlier-stage sarcopenia where compensatory mechanisms preserve muscle function but fail to maintain bone integrity.

Based on our findings, we recommend that sarcopenia screening should be integrated into osteoporosis risk assessments. Since sarcopenia is significantly associated with an increased risk of osteoporosis, early identification of sarcopenia can help clinicians identify patients at high risk of osteoporosis. This can enable timely interventions such as exercise programs, nutritional supplements, and lifestyle modifications to prevent or delay the development of osteoporosis. Clinicians should also be aware of the differences in risk among different populations (such as gender, geographical regions) when assessing osteoporosis risk.

Importantly, consistent risk estimates across diagnostic criteria (e.g., EWGSOP vs. AWGS) highlight the cross-cultural stability of the muscle-bone density relationship. These findings reinforce the integrative bone-muscle axis, where mechanical loading, endocrine signaling, and paracrine networks coordinate mass homeostasis, and suggest that early sarcopenia intervention may mitigate osteoporosis risk in aging populations.

Sarcopenia and osteoporosis exhibit significant pathophysiological overlap, with accumulating evidence indicating shared regulatory pathways and bidirectional interactions (20). As integral components of the locomotor system, bone and muscle demonstrate coordinated mass changes across the lifespan, orchestrated by a triad of mechanical loading, endocrine regulation, and paracrine signaling networks (21). Notably, exercise deprivation, disuse atrophy, and aging-induced catabolism trigger synchronous bone-muscle degeneration, as posited by the Mechanical Homeostasis Theory, where reduced mechanical stimuli disrupt bone anabolic processes, leading to microarchitectural deterioration (22, 23). Within this framework, skeletal muscle serves as the primary transducer of mechanical loading, providing critical anabolic signals for bone maintenance.

While mechanical coupling is well-established, the systemic mechanisms governing bone-muscle mass equilibrium remain incompletely understood. Emerging evidence highlights secreted factors as key mediators: signaling molecules including myostatin, activin, and pro-inflammatory cytokines reciprocally regulate bone and muscle metabolism (24, 25). Skeletal muscle has recently been redefined as an endocrine organ, secreting myokines that not only modulate glucose metabolism but also form regulatory networks with osteokines, providing novel insights into integrated bone-muscle physiology.

At the molecular level, the Wnt/β-catenin signaling pathway emerges as a dual regulator, governing both bone mass homeostasis and skeletal muscle development (25). Osteocyte-derived sclerostin (encoded by SOST), a potent Wnt antagonist, acts as a pivotal node in this cross-talk, suggesting osteocytes establish biochemical dialogues with muscle tissues through secreted factors.

In summary, sarcopenia confers a significant osteoporosis risk, emphasizing the need for integrated screening strategies. Future longitudinal studies are required to clarify causal relationships and identify novel therapeutic targets within the bone-muscle metabolic axis.

In conclusion, sarcopenia is associated with an increased risk of osteoporosis, and early prevention of osteoporosis may focus on better identification and prevention of sarcopenia. In the future, more high-quality longitudinal studies are needed to provide insight into the correlation and potential mechanisms between sarcopenia and osteoporosis.

## Data Availability

The original contributions presented in this study are included in this article/supplementary material, further inquiries can be directed to the corresponding author.
